# A mouse model for functional dissection of TAB1 O-GlcNAcylation

**DOI:** 10.12688/wellcomeopenres.15394.2

**Published:** 2020-06-16

**Authors:** Florence Authier, Villő Muha, Daan M.F. van Aalten

**Affiliations:** 1 Division of Gene Regulation and Expression, School of Life Sciences, University of Dundee, Dundee, DD1 5EH, UK

**Keywords:** O-GlcNAcylation, TAB1, TAK1 signalling, inflammation

## Abstract

**Background:** O-GlcNAcylation is a posttranslational modification associated with various physiological and pathophysiological processes including diabetes, cancer, neurodegeneration and inflammation. However, the biological mechanisms underlying the role of specific O-GlcNAc sites and their link to phenotypes remain largely unexplored due to lack of suitable *in vivo* models. TGF-β activated kinase-1 binding protein-1 (TAB1) is a scaffolding protein required for TGF-β activated kinase-1 (TAK1) mediated signalling. A single O-GlcNAc site has been identified on human TAB1 that modulates TAK1-mediated cytokine release in cells.

**Methods:** Here, we report the generation of the *Tab1 ^S393A ^*mouse model using a constitutive knock-in strategy. The *Tab1 ^S393A ^*mice carry a Ser393Ala (S393A) mutation that leads to loss of O-GlcNAcylation site on TAB1.

**Results: **We did not observe any obvious phenotype in *Tab1 ^S393A^* mice. Loss of O-GlcNAcylation on TAB1 has no consequences on TAB1 protein level or on TAB1-TAK1 interaction.

**Conclusions:** The homozygous *Tab1 ^S393A ^*mice are viable and develop with no obvious abnormalities, providing a powerful tool to further investigate the role of O-GlcNAc on TAB1 in the inflammatory response in the context of a whole organism.

## Introduction

O-GlcNAcylation is a dynamic and reversible post-translational modification on serine and threonine residues of intracellular proteins. It is catalysed by two enzymes, O-linked N-acetylglucosaminyltransferase (OGT) and β-N-acetylglucosaminidase (O-GlcNAcase, OGA), responsible for the addition and removal of the O-GlcNAc group, respectively ( Hart *et al.,* 2011). O-GlcNAcylation modulates several cellular processes, such as transcription ( Hanover *et al.,* 2012), cell signalling ( Muller *et al.,* 2013; Zachara *et al.,* 2004), and metabolism ( Ruan *et al.,* 2013). Changes in O-GlcNAc levels are associated with pathological conditions including diabetes, neurodegeneration and cancer ( Issad *et al.,* 2010; Lazarus *et al.,* 2009; Slawson *et al.,* 2010). In addition, accumulating evidence suggests that O-GlcNAc modification controls the maturation and activation of immune cells, including T cells, B cells and macrophages ( Golks & Guerini, 2008; Li *et al.,* 2019; Machacek *et al.,* 2019).

O-GlcNAcylation has been shown to affect the transforming growth factor (TGF)-β-activated kinase 1 (TAK1) signalling pathway ( Mendoza *et al.,* 2008). The TAK1 pathway regulates inflammatory cytokine production and release in response to pro-inflammatory and endotoxin stimuli in macrophages and innate immune cells. TAK1 forms a functional complex with the pseudo-phosphatase TAK1 binding protein-1 (TAB1) and regulates the production of inflammatory molecules through activation of several mitogen-activated protein kinases (MAPKs) including p38α, extracellular signal-regulated kinases (ERKs) and c-jun kinases, leading to subsequent activation of downstream effectors including the IκB kinases (IKKs) and the transcription factor NFκB ( Adhikari *et al.,* 2007; Conner *et al.,* 2006; Sato *et al.,* 2005; Shibuya *et al.,* 1996; Shim *et al.,* 2005; Wang *et al.,* 2001). TAB1 is required for TAK1 activation and downstream signalling events. TAK1 activity can be negatively regulated by p38α MAPK through a feedback mechanism where p38α MAPK phosphorylates TAB1, leading to TAK1 activity suppression ( Cheung *et al.,* 2003). The TAK1-TAB1 complex has been found to be important *in vivo* for the survival of activated macrophages upon lipopolysaccharide (LPS) stimulation and for the modulation of immune response in T and B cells ( Mihaly *et al.,* 2014; Sato *et al.,* 2005).

We previously showed that human TAB1 is modified with N-acetylglucosamine (O-GlcNAc) on a single site, Ser395, in human cells. O-GlcNAcylation of TAB1 is induced under stress conditions and modulates TAK1-mediated cytokine release *in vitro* by increasing TAK1 activation, leading to downstream signalling activation and cytokine production in mouse embryonic fibroblasts (MEFs) ( Pathak *et al.,* 2012). However, the *in vivo* biological significance of this single O-GlcNAc site remains to be explored.

Here, we describe the generation of a genome edited constitutive knock-in (KI) mouse model expressing endogenous TAB1 lacking the key residue targeted for O-GlcNAcylation. The Ser393 site, equivalent to Ser395 in the human TAB1 sequence, was abolished by introducing a Ser393Ala mutation using a classical recombinational approach. We demonstrate that this mutation causes the loss of the O-GlcNAc modification on TAB1 without affecting TAB1 protein levels or its interaction with TAK1. Homozygous *Tab1 ^S393A^* mice lacking O-GlcNAc on TAB1 were viable with no obvious development abnormalities. This work provides a platform for exploration of O-GlcNAc-dependent functions of TAB1.

## Results

### Generation of *Tab1 ^S393A^* KI mice

**Figure 1.**
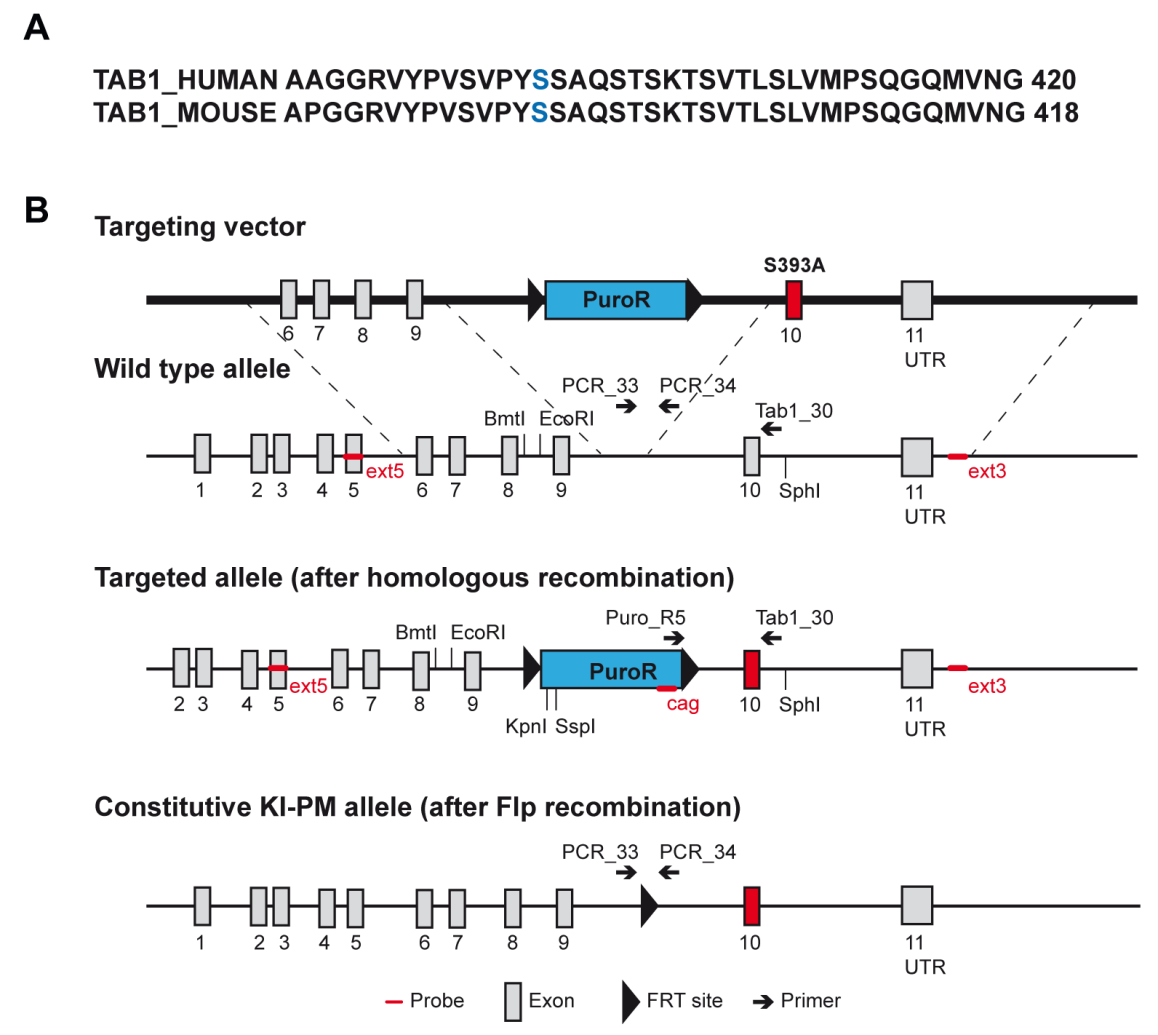
Targeting strategy for the generation of constitutive *Tab1 ^S393A^* knock-in (KI) mice. ( **a**) Sequence alignment of human (UniProt Q15750) and mouse (UniProt Q8CF89) TAB1 covering the 382-420aa of the C-terminal domain. The human and mouse O-GlcNAc sites are reported in blue. ( **b**) Schematic representation of the targeting vector containing a flippase recognition target (FRT)-flanked puromycin resistance cassette in intron 9, wild type allele, targeted allele after homologous recombination and the constitutive KI point mutation (PM) allele after flippase (Flp) recombination. The locations of the enzymatic restriction sites, primers and probes are also shown.

**Figure 2.**
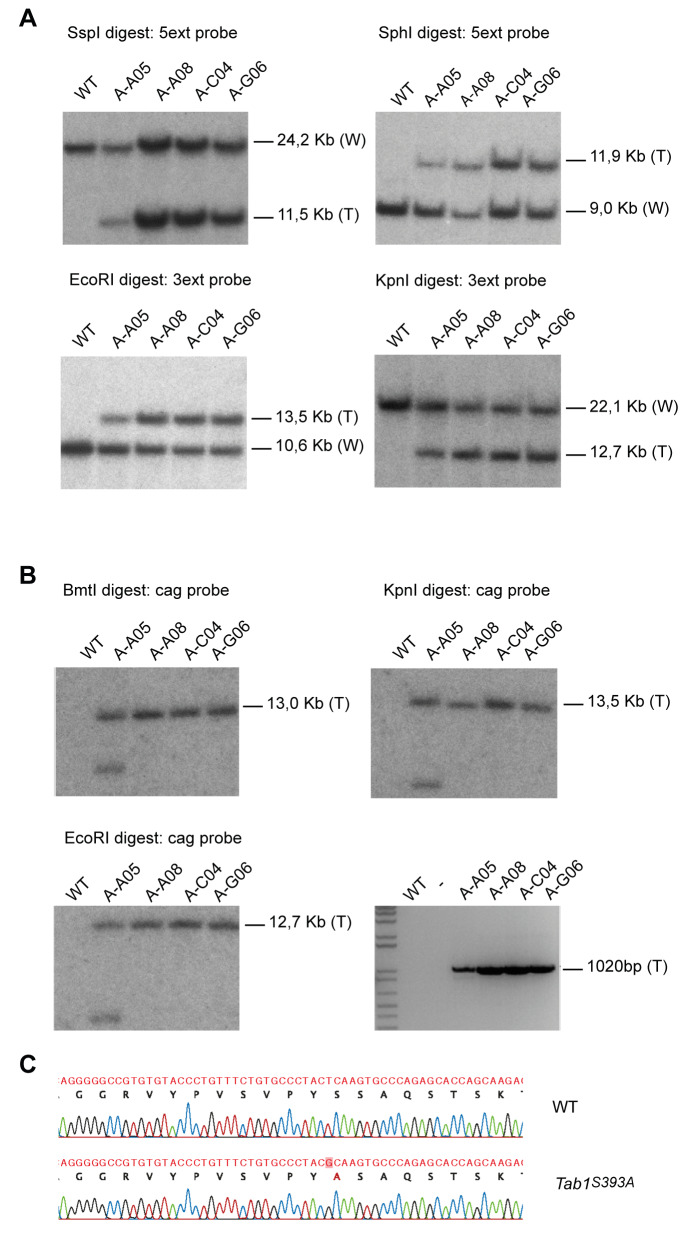
Generation of correctly targeted embryonic stem (ES) clones bearing the constitutive knock-in (KI) S393A point mutation in TAB1. ( **a**) Southern blot of wild-type (WT) and four targeted ES clones (named A-A05, A-A08, A-C04 and A-G06) with 5’ ext and 3’ ext probes shows correct homologous recombination at the 5’ and 3’ sides in all clones except for A-C05.The expected molecular weight band for WT (W) and the targeted (T) alleles are shown. ( **b**) Southern blot of WT and four targeted ES clones (named A-A05, A-A08, A-C04 and A-G06) with the cag probe shows single integration in all clones except for A-C05 (upper left and right panels and lower left panel). The expected molecular weight band for the targeted (T) allele is shown. PCR of WT and four targeted ES clones (named A-A05, A-A08, A-C04 and A-G06) shows insertion of the point mutation in all targeted clones (lower right panel). The expected molecular weight band for the targeted (T) allele is shown. ( **c**) Sequencing of the genomic DNA from WT and KI *Tab1 ^S393A^* mice confirming the S393A single point mutation (in red) in KI animal.

We previously showed that human TAB1 is modified with N-acetylglucosamine (O-GlcNAc) on a single site, Ser395 ( Pathak *et al*., 2012). TAB1 sequence is 97% identical between human and mouse. The O-GlcNAc modified Ser395 residue in human is conserved in mice and corresponds to the Ser393 ( Figure 1A). The Ser393 residue is the sole reported O-GlcNAc site on mouse TAB1 ( Alfaro *et al*., 2012; Trinidad *et al*., 2012). To establish an animal model for investigating the role of O-GlcNAc on TAB1 *in vivo*, we generated TAB1 constitutive knock-in (KI) mice carrying a Ser393Ala (S393A) mutation. The *Tab1* gene is located on mouse chromosome 15 and contains 11 exons. The nucleotide sequence encoding the amino acid S393 region is located on exon 10. The targeting vector contained the translation initiation codon in exon 1 and an FRT-flanked puromycin cassette in the intronic sequence between exons 9 and 10. The targeted allele was obtained via homologous recombination in C57Bl/6NTac ES cells. The constitutive KI-point mutation (PM) allele was obtained after *in vivo* Flp recombination ( Figure 1B). Positive targeted ES cell clones were confirmed by Southern blot analysis. Correct homologous recombination at 5’ and 3’ sides was detected in A-A08, A-C04, B-G06 clones ( Figure 2A). A single integration site was also confirmed by Southern blot analysis using the cag probe, which detects a region located within the CAG_PuroR_pA selection cassette. Correct single integration was detected in A-A08, A-C04, B-G06 clones ( Figure 2B upper panels and left lower panel). The insertion of the point mutation was validated by PCR genotyping and detected in all the targeted clones ( Figure 2B right lower panel). The A-A08 and A-C04 positive clones were injected into BALB/c blastocysts to generate chimeric mice. Genotyping was performed to identify constitutive KI animals. The presence of the S393A mutation was confirmed by sequencing ( Figure 2C). Heterozygous mice were backcrossed to C57BL/6J genetic background and maintained as heterozygotes. Homozygous *Tab1 ^S393A^* mice were obtained by breeding heterozygous offspring. Thus, we successfully generated TAB1 S393A KI mice lacking the O-GlcNAc site at Ser393.

### *Tab1 ^S393A^* KI mice exhibit normal development and survival

**Figure 3.**
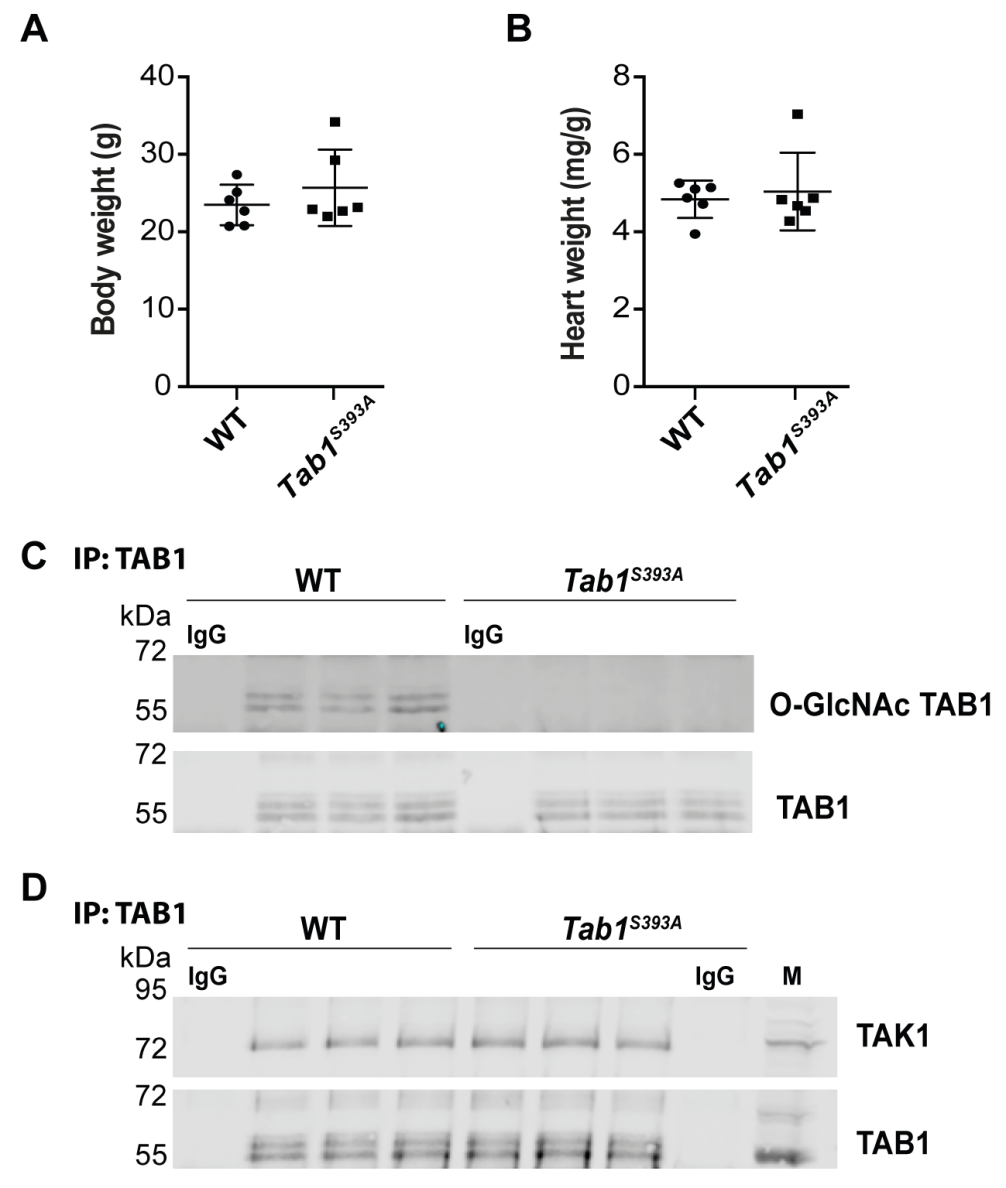
*Tab1 ^S393A^* knock-in (KI) mice lacking O-GlcNAcylation on TAB1 exhibit normal development and retained TAB1-TAK1 interaction. ( **a**) Body weight of wild-type (WT) and *Tab1 ^S393A^* KI at three months old. Data are expressed as the mean ± SD (WT, n=6; *Tab1 ^S393A^*, n=6), Student’s t-test. ( **b**) Heart weight per g of body weight of WT and *Tab1 ^S393A^* KI at three months old. Data are expressed as the mean ± SD (WT, n=6; *Tab1 ^S393A^*, n=6), Student’s t-test. ( **c**) TAB1 protein immunoprecipitation (IP) and blotting using anti-gTAB1 and anti-TAB1 antibodies in brain tissues of WT and *Tab1 ^S393A^* mice shows loss of O-GlcNAcylation of TAB1 in *Tab1 ^S393A^* compared to WT animals with similar TAB1 protein levels in both genotypes (WT, n=3; *Tab1 ^S393A^*, n=3). ( **d**) TAB1 protein immunoprecipitation (IP) and blotting using anti-TAK1 and anti-TAB1 antibodies in brain tissues of WT and *Tab1 ^S393A^* mice shows retention of TAB1-TAK1 interaction in both genotypes (WT, n=3; *Tab1 ^S393A^*, n=3). IgG: Isotype control; M: mouse macrophage lysate.

We used *Tab1 ^S393A/+^* heterozygous animals to generate homozygous *Tab1 ^S393A^* animals. We obtained 19 WT, 68 *Tab1 ^S393A/+^* and 17 *Tab1 ^S393A^* animals from heterozygous pairs. Although deletion of TAB1 leads to embryonic lethality and abnormal cardiovascular development in mice ( Komatsu *et al.,* 2002), the homozygous *Tab1 ^S393A^* mice lacking O-GlcNAc on TAB1 were viable and developed normally. The *Tab1 ^S393A^* mice appeared healthy and similar to WT animals. They showed comparable body weight and heart size to WT at three months of age ( Figure 3A and 3B; Authier, 2019b). In addition, newborn pups were obtained by breeding of either male or female homozygous *Tab1 ^S393A^* mice with sex-matched WT animals, suggesting that fertility is not altered in these animals.

TAB1 is ubiquitously expressed in mammals ( Komatsu *et al.,* 2002; Shibuya *et al.,* 1996). To verify that the S393A mutation leads to absence of O-GlcNAcylation of TAB1 in the transgenic animals, brain tissues were isolated from WT and *Tab1 ^S393A^* mice and TAB1 was immunoprecipitated prior to probing with a site specific O-GlcNAc TAB1 antibody ( Pathak *et al.,* 2012). As expected, TAB1 was found glycosylated in WT animals, while the S393A mutation led to absence of O-GlcNAc on TAB1 in *Tab1 ^S393A^* mice ( Figure 3C).

We next investigated the impact of the S393A mutation on TAB1 protein levels. We observed similar levels of TAB1 protein expression in WT and *Tab1 ^S393A^* animals suggesting that absence of O-GlcNAc of TAB1 has no detectable influence on TAB1 protein levels ( Figure 3C). Thus, Tab1 ^*S393A*^ KI mice lacking O-GlcNAc on TAB1 exhibit normal development and survival.

### O-GlcNAcylation of TAB1 is not required for its interaction with TAK1

TAB1 is found in complex with TAK1 and this interaction is required for TAK1-mediated signalling ( Mendoza *et al.,* 2008). To investigate the effect of loss of O-GlcNAcylation on TAB1 on the interaction with TAK1, immunoprecipitation analysis was performed on brain tissue lysates from WT and *Tab1 ^S393A^* mice. We found that TAB1 interacts with TAK1 in both WT and transgenic animals, suggesting that O-GlcNAcylation of TAB1 is not required for its interaction with TAK1 ( Figure 3D).

## Discussion

Although O-GlcNAcylation was discovered over 30 years ago, our knowledge on the biological processes regulated by site-specific O-GlcNAcylation of specific proteins is limited. Generation of transgenic animal models lacking specific O-GlcNAc sites will help to decipher the role of individual O-GlcNAc sites and associated mechanisms in physiological and pathologic conditions. In the present study we have described the generation of a viable constitutive KI mouse model leading to loss of the sole reported O-GlcNAc site S393 on TAB1 protein, allowing for determining the biological significance of TAB1 O-GlcNAcylation *in vivo*.

A growing body of evidence supports that O-GlcNAcylation is an important modulator of the immune response and inflammation ( Golks & Guerini, 2008; Golks *et al.,* 2007; Kearse & Hart, 1991). However, the role of this modification on specific proteins involved in the function and regulation of immune cells remains largely unknown. The TAB1-TAK1 complex regulates the activation, survival and maintenance of activated macrophages upon LPS stimulation *in vivo* ( Mihaly *et al.,* 2014). O-GlcNAcylation of TAB1 modulates TAK1-mediated cytokine release in response to IL-1 stimulation or osmotic stress in MEF cells ( Pathak *et al.,* 2012). However, these pathways have not been investigated *in vivo*. The *Tab1 ^S393^* mice model will allow investigation of the consequences of the loss of O-GlcNAc of TAB1 on TAK1 activation and signalling in the context of a whole organism.

TAB1 is also involved in a negative feedback loop in which p38α MAPK suppresses TAK1 activation through phosphorylation of TAB1 at two residues; Ser423 and Thr431 ( Cheung *et al.,* 2003). The p38α MAPK is a protein serine-threonine kinase involved in signalling pathways induced by stress. It is activated by transphosphorylation of the TGY motif by MAPK kinases (MKKs) 3, 4 and 6 ( Enslen *et al.,* 1998; Raingeaud *et al.,* 1996). Besides being a substrate for p38α MAPK, TAB1 binds to p38α, leading to its autophosphorylation of Thr180 and Tyr182 residues ( Ge *et al.,* 2002). This mechanism constitutes an alternative activation pathway for p38α MAPK that has been demonstrated to be independent to the MKK cascade and does not lead to classical p38α MAPK downstream signalling and inflammatory gene expression ( Lu *et al.,* 2006). The autoactivation of p38α MAPK through TAB1 was observed in pathological conditions, including cardiac ischemia and heart failure and has been shown to aggravate myocardial injury ( De Nicola *et al.,* 2018; Li *et al.,* 2005; Shi *et al.,* 2010; Tanno *et al.,* 2003). A recent study has demonstrated that the p38α-TAB1 interaction can be targeted by small molecules ( De Nicola *et al.,* 2018). The same study reported that reduction of infarction volume was observed in a KI mouse model in which residues located in the p38α docking region of TAB1 were mutated to prevent the p38α-TAB1 interaction ( De Nicola *et al.,* 2018). Thus, the development of strategy to target the TAB1-p38α MAPK may be therapeutically relevant. Interestingly, the single O-GlcNAc site of TAB1 is located within this p38α MAPK binding region ( DeNicola *et al.,* 2013; Pathak *et al.,* 2012), which is required for p38α MAPK activation ( Ge *et al.,* 2002). It is possible that O-GlcNAc on TAB1 could play a role in modulating the TAB1-p38α interaction and subsequent p38α MAPK autoactivation. In the future, both *in vitro* and *in vivo* experiments could be performed to test this hypothesis. *Tab1 ^S393A^* mice lacking O-GlcNAc on TAB1 will provide a suitable model to investigate the potential effect of this posttranslational modification on p38α MAPK signalling during cardiac injury in mice.

In our study, we did not observe any obvious phenotype in *Tab1 ^S393A^* mice. Given the role of TAB1 in immune response and its interactors, it is likely that induction of stress as inflammation could reveal potential phenotypes. We conclude that this model could be a valuable tool to further investigate the functional relevance of the sole reported O-GlcNAcylation site of mouse TAB1 in a whole organism at several stages of development but also in pathological conditions.

## Methods

### Ethical statement

All efforts were made to ameliorate any suffering of animals by providing appropriate husbandry environment conditions (listed below) in accordance with UK and European Union regulations. All animal studies were conducted in accordance with the Animal (Scientific Procedures) Act 1986 for the care and use of laboratory animals, and procedures were carried out under United Kingdom Home Office regulation and the project licence PAAE38C7B with approval by the Welfare and Ethical Use of Animals Committee of University of Dundee. Mice were humanely killed using a Schedule 1 method as soon as any sign of distress or suffering were observed.

### Animal source and husbandry

Mice were maintained on a C57BL/6J (Charles River UK) background and kept in groups of five animals in individually ventilated cages at 21°C, 45–65% relative humidity and a 12h/12h light/dark cycle under specific-pathogen–free conditions in accordance with UK and European Union regulations. Mice had access to a mouse house with sizzle-nest material for bedding and corn cob for nesting and free access to food (R&M3 pelleted irradiated diet) and 0.2 micron sterile filtered water.

### Generation of TAB1 constitutive knock-in S393A mice

The TAB1 knock-in (KI) mouse line (C57BL/6NTac-Tab1 ^*tm3593(S393A)Arte*^) carrying a S393A mutation was generated by Taconic Artemis GmbH using the targeting strategy illustrated in Figure 1 and based on the NCBI transcript NM_025609_2. The targeting vector contains the translation initiation codon in exon 1 and also contains a flippase recognition target (FRT)-flanked puromycin cassette in intron 9. The targeting vector was generated using bacterial artificial chromosome (BAC) clones from the C57BL/6J RPCIB-731 Tac BAC library and transfected by electroporation into C57BL/6NTac embryonic stem (ES) cell lines. Recombinant ES cell clones were identified by Southern blot using external (5’ ext and 3’ ext probes) and internal probes (cag). The genomic DNA of the ES cell clones were digested with *Ssp*I or *Sph*I for the 5’ ext probe and analysed for 11.5 kb ( *Ssp*I digest) or 11.9 kb ( *Sph*I digest) fragments for the recombinant allele, in addition to 24.2 kb ( *Ssp*I digest) or 9 kb ( *Sph*I digest) for the wild-type (WT) allele. For the 3’ ext probe, the genomic DNA was digested with *Eco*RI or *Kpn*I and analysed for 13.5 kb ( *Eco*RI digest) or 12.7 kb ( *Kpn*I digest) fragments for the recombinant allele, and 10.6 kb ( *Eco*RI digest) or 22.1 kb ( *Kpn*I digest) fragments for the WT allele. Single integration was also confirmed by Southern blot analysis using the cag probe that detects a region located within the puromycin selection cassette. The genomic DNA of the ES cell clones was digested with *Bmt*I, *Eco*RI or *Kpn*I restriction enzymes and analysed for single fragments of 13.0 kb ( *Bmt*I digest), 13.5 kb ( *Eco*RI digest) or 12.7 kb ( *Kpn*I digest) for the recombinant allele. The insertion of the point mutation S393A was validated by PCR genotyping using the Puro_R5 and Tab1_30 primers, recombinant Taq DNA polymerase (EP0402, Invitrogen) and T100 Thermal Cycler (Biorad). The following PCR program was used (98 °C for 2 min, 98 °C for 10 sec, 68 °C for 1 min, 40 cycles, 20 °C for 10 min). Two positive targeted ES cell clones, A-A08 and A-C04, were injected using piezo actuated microinjection pipette with an internal diameter of 12–15 μm into blastocyst cells from 10 superovulated 4/5 weeks old BALB/c female mice (BALB/cAnNTac, Taconic Artemis GmbH), which were later transferred into six pseudopregnant albino NMRI females (BomTac:NMRI, Taconic Artemis GmbH) and 31 chimeric mice were obtained. Chimerism was measured by coat colour contribution of ES cells to BALB/c host (black/white). Based on visual estimation, three highly chimeric (>75%) seven-week-old male mice were selected for further breeding with six age-matched flippase (Flp) deleter C57BL/6 female mice (C57BL/6- *Tg(CAG-Flpe)2 Arte,* Taconic Artemis GmbH) to remove the selection marker. From this breeding, 28 mice reached the weaning stage and were used for mouse genotyping analysis. Genomic DNA was extracted from ear notches collected during the mice identification procedure using an ear punch tool (Braintree Scientific, Inc) and analysed by PCR using recombinant Taq DNA polymerase (EP0402, Invitrogen) and T100 Thermal Cycler (Biorad). The PCR_33 and PCR_34 primers amplified bands of 259 bp and 334 bp for the WT and the constitutive KI, respectively, using the following PCR program (95 °C for 5 min, 95 °C for 30 sec, 60 °C for 30 sec, 72 °C for 1 min, 35 cycles, 72 °C for 10 min). The presence of the point mutation S393A was validated by DNA sequencing using the 10597_35 and 10597_36primers DNA amplification and the MT93_dig_seq for the sequencing analysis with Applied Biosystems 3730 device (ThermoFisher Scientific). The primers sequences used were: 5’ ext probe sense: AAGCTAAGGTGGCCCTCAAGCAC, 5’ ext probe antisense: CACCGACATTGGCAACGTAGAG, 3’ ext probe sense: TCTAAGGTCAGTGCCTATCATACG, 3’ ext probe antisense: GGACTTAACCTCTCGCTGTAAGACC, Puro_R5: CCGTAAGTTATGTAACGCGGAACTCC, Tab1_30: ATTGGAGGCCAAAGATCCAGAGTCC, PCR_33: TCACTTCTCACCCTTACCAGC, PCR_34: AATGGGAGGTGAGAGCTCC, 10597_35: AGGCCACTACCTTGGTTTCC, 10597_36: CTGTGTCTAATAAGACTCCCTACCC, MT93_dig_seq: TGGAGAATTGGAGGCCAAAG.

### Body weight measurement and tissue collection

Heterozygous offspring were used for breeding to generate homozygous *Tab1 ^S393A^* mice. WT and homozygous *Tab1 ^S393A^* mice were housed together in the same cage in groups of five animals. Body weight of six WT mice (three female and three male) and six homozygous *Tab1 ^S393A^* mice (four female and two male) were measured at three months of age. Data collection was performed by an independent observer in a blinded manner. For tissue collection, mice were euthanized by carbon dioxide gas exposure in a rising concentration and brain and heart tissue samples were collected, weighted and rapidly snap frozen in liquid nitrogen.

### Immunoprecipitation of TAB1 and immunoblotting

Endogenous TAB1 was immunoprecipitated as described previously ( Pathak *et al.,* 2012). Briefly, mouse brain tissue from three WT and three homozygous *Tab1 ^S393A^* mice was lysed in 50 mM Tris-HCl pH 7.4 0.1 mM EGTA, 1 mM EDTA, 1 % Triton-X100, 1 mM sodium orthovanadate, 50 mM sodium fluoride, 5 mM sodium pyrophosphate, 0.27 M sucrose, 0.1 % 2-mercaptoethanol supplemented with protease inhibitors (1 mM benzamidine, 0.2 mM PMSF and 5 μM leupeptin) and 10 μM GlcNAcstatin G. Brain lysate was centrifuged with 14,000 rpm for 20 minutes at 4 **°**C and the protein concentration was determined with Pierce 660 nm protein assay (22660, Thermofisher Scientific). Cleared brain lysate containing 1 mg of input protein was incubated for two hours at 4 °C with 10 μg of anti-TAB1 (sheep polyclonal S823A, Division of Signal Transduction Therapy, DSTT, University of Dundee) or anti-IgG antibody coupled with 10 μl of Dynabeads Protein G (10009D, Thermofisher). The beads were washed twice with 1 ml of lysis buffer containing 0.25 M NaCl, followed by two washes with 1 ml of 50 mM Tris/HCl, pH 7.5, 50 mM NaCl and 0.1 % (v/v) 2-mercaptoethanol. Eluted samples were subjected to sodium dodecyl sulfate polyacrylamide gel electrophoresis (SDS–PAGE) followed by Western blotting ( Pathak *et al.,* 2012). Membranes were incubated with primary antibodies specific to in-house produced gTAB1 (rabbit polyclonal, 1:1000, University of Dundee) ( Pathak *et al.,* 2012), TAB1 (sheep polyclonal S823A, 1:1000, DSTT) and TAK1 (sheep polyclonal S828A, 1:1000, DSTT) in blocking buffer, 5 % BSA in TBST (Tris-buffered saline with 0.1 % Tween-20) overnight at 4 °C and next with IR680 (catalogue # 925-68024, Li-Cor) and IR800 (RRID: AB_621848, catalogue # 926-32213, Li-Cor) donkey labelled secondary antibodies at room temperature for one hour. Blots were imaged using Li-Cor Odyssey infrared imaging system (Li-Cor). Original unedited gels and details of the controls used can be found here ( Authier, 2019a).

### Statistical analysis

Statistical analyses were performed with GraphPad Prism version 6 software. For pairwise comparisons of WT and *Tab1 ^S393A^* body weight and heart size data, the Student’s t-test was used.

## Data availability

### Underlying data

Figshare: Original unedited gels. https://doi.org/10.6084/m9.figshare.9206567.v1 ( Authier, 2019a)

Figshare: Body weight and heart weight raw data. https://doi.org/10.6084/m9.figshare.9206546 ( Authier, 2019b)

Data are available under the terms of the Creative Commons Attribution 4.0 International license (CC-BY 4.0).
