# Supplementary material for: A mouse model for functional dissection of TAB1 O-GlcNAcylation
Source: Wellcome Open Res. 2020 Jun 16;4:128. Originally published 2019 Aug 27. [Version 2] doi: 10.12688/wellcomeopenres.15394.2 (PMC7333360; doi:10.12688/wellcomeopenres.15394.2)
Supplement: Peer review history

# Peer review: Reviewer response for version 2

**DOI:** 10.21956/wellcomeopenres.17534.r39127

**Published:** 2020-07-02

**Authors:** Kuo-I Lin

**Affiliations:** 1 Genomics Research Center, Academia Sinica, Taipei, Taiwan

Is the work clearly and accurately presented and does it cite the current literature?

Yes

If applicable, is the statistical analysis and its interpretation appropriate?

Not applicable

Are all the source data underlying the results available to ensure full reproducibility?

Yes

Is the study design appropriate and is the work technically sound?

Partly

Are the conclusions drawn adequately supported by the results?

Partly

Are sufficient details of methods and analysis provided to allow replication by others?

Yes

Reviewer Expertise:

NA

I confirm that I have read this submission and believe that I have an appropriate level of expertise to confirm that it is of an acceptable scientific standard.

# Peer review: Reviewer response for version 1

**DOI:** 10.21956/wellcomeopenres.16824.r36480

**Published:** 2019-10-07

**Authors:** Gian F. De Nicola

**Affiliations:** 1 British Heart Foundation Centre of Excellence, The Rayne Institute, St. Thomas’ Hospital, King's College London, London, UK

Authier et al. in their manuscript characterise a new knock-in mouse with a Serine to Alanine mutation on residue 393 of TAB1. This leads to a loss of the only O-GlcNAcylation site on TAB1. They confirm the loss of O-GlcNAcylation using an in house site specific anti g-TAB1 antibody.They go on to characterise the mice at baseline where they do not observe any obvious phenotype. 

 This new mouse model will be a valuable tool to investigate the role of GlcNAcylation in MAPK signalling in homeostasis and pathological settings.

 The manuscript is a brief report on a preliminary characterisation of a new knock-in mouse model at baseline, the characterisation is quite limited and there could be few more experiments that could expand the characterisation but I am not sure whether they would go beyond the scope of the journal.

 For instance:

1. it would be interesting to measure protein and mRNA levels of TAB1 in few more tissues and organs to test if O-GlcNAcylation has any role in protein stability.
2. TAB1 knock-out is embryonic lethal, in KOs, heart development and myocardial cell differentiation are impaired, it would be therefore useful to compare basic heart functions (heart rate, blood pressure, LV ejection fraction) between KI and WT at baseline, it could reveal a subtle difference that is exacerbated upon stress.
3. TAB1 is both a substrate and activator of p38alpha which sits downstream of TAK1. It would be interesting to compare the levels of p38alpha and TAB1 phosphorylation to test whether TAB1 ser 393 O-GlcNAcylation affects TAK1 signalling at baseline.

 The authors observe that it is likely that the induction of stresses such as inflammation or ischaemia might reveal potential phenotypes. Testing such hypothesis would be of great interest but probably as I mentioned it goes beyond the scope of this journal, nevertheless this new mouse model is an useful tool to investigate the biology of TAB1 O-GlcNAcylation.

Is the work clearly and accurately presented and does it cite the current literature?

Yes

If applicable, is the statistical analysis and its interpretation appropriate?

Yes

Are all the source data underlying the results available to ensure full reproducibility?

Yes

Is the study design appropriate and is the work technically sound?

Yes

Are the conclusions drawn adequately supported by the results?

Yes

Are sufficient details of methods and analysis provided to allow replication by others?

Yes

Reviewer Expertise:

MAPK signalling. protein-protein inhibitors

I confirm that I have read this submission and believe that I have an appropriate level of expertise to confirm that it is of an acceptable scientific standard.

# Response

**Published:** 2020-06-11

**Authors:** Daan van Aalten

**Affiliations:** University of Dundee, UK

We thank the referees for their critical reading of the manuscript and constructive feedback. Several points for improving the manuscript were made, in addition to good suggestions for further studies. Our aim of publishing this work in a rapid open access journal such as Wellcome Open Research was to report the availability of this mouse model, facilitating future research to dissect the role of this specific O-GlcNAc site on TAB1. As such, most of the experiments proposed are outside the scope of this manuscript and it is hoped our model will allow such experiments to be conducted in the future.

 Reviewer 2:

 The manuscript is a brief report on preliminary characterisation of a new knock-in mouse model at baseline, the characterisation is quite limited and there could be few more experiments that could expand the characterisation but I am not sure whether they would go beyond the scope of the journal.

 For instance:

1. it would be interesting to measure protein and mRNA levels of TAB1 in few more tissues and organs to test if O-GlcNAcylation has any role in protein stability.
2. TAB1 knock-out is embryonic lethal, in KOs, heart development and myocardial cell differentiation are impaired, it would be therefore useful to compare basic heart functions (heart rate, blood pressure, LV ejection fraction) between KI and WT at baseline, it could reveal a subtle difference that is exacerbated upon stress.
3. TAB1 is both a substrate and activator of p38alpha which sits downstream of TAK1. It would be interesting to compare the levels of p38alpha and TAB1 phosphorylation to test whether TAB1 ser 393 O-GlcNAcylation affects TAK1 signalling at baseline.

We agree with the referee that this mouse model now enables a number of hypothesis as to the role of TAB1 O-GlcNAcylation to be tested, but as the referee suggests, this paper is focused on describing the generation of the model, and demonstration of loss of O-GlcNAcylation. The experiments the referee proposes are excellent suggestions, but beyond the scope of this manuscript.

# Peer review: Reviewer response for version 1

**DOI:** 10.21956/wellcomeopenres.16824.r36321

**Published:** 2019-09-19

**Authors:** Kuo-I Lin

**Affiliations:** 1 Genomics Research Center, Academia Sinica, Taipei, Taiwan

This manuscript described a mouse model for studying the function of O-GlcNAcylation of TAB1 by generating a conventional knock-in (KI) mouse in which the single O-GlcNAcylation site at TAB1 Ser 393 was mutated to Ala ( *Tab1* ^S393A^). The homozygous mutation mice were viable and had no obvious abnormalities. The authors further showed that loss of O-GlcNAcylation of TAB1 did not affect TAB1 protein level or interaction with TAK1. The authors concluded that this *Tab1* ^S393A ^KI mouse line could be a good tool to study the role of O-GlcNAcylation on TAB1 in inflammation responses. Overall, this knock-in mouse line can be a potentially useful tool to study the importance of O-GlcNAcylation on TAB1 in inflammation. However, there are some major points need to be clarified and addressed:

1. Although the information was described in the Methods, the authors should also indicate the location of the restriction enzyme sites, primers and probes used in Figure 1. This will help the readers to understand the strategy. The authors should also provide the raw data of genomic sequencing containing the sequences encoding 393 Ala in KI and 393 Ser in WT mice.
2. It has been reported that Tab1-deficiency caused embryonic lethality, due to the defects in lung and cardiovascular morphogenesis. ^1^ The authors showed that the heart weight of *Tab1* ^S393A ^mouse and WT mouse is comparable (Figure 3). It is unclear if there are any morphological abnormalities in the heart and lung of *Tab1* ^S393A ^mice. Histological analyses of heart and lung tissues should be done here. In addition, Figure 3B shows the heart weight is comparable between *Tab1* ^S393A ^and WT mouse at three months old. The unit in Y-axis should be clarified.
3. The title of this report is "A mouse model for functional dissection of TAB1 O-GlcNAcylation". To convince the readers that this mouse model can be used to dissect the function of TAB1 O-GlcNAcylation, the authors should demonstrate the potential biological effects of losing O-GlcNAcylation on TAB1 Ser393.  
4. In Figure 3C, the authors used site-specific antibody to detect TAB1 O-GlcNAcylation in mouse cells. Although it has been reported that human TAB1 is O-GlcNAcylated on a single site at Ser395 (which is equivalent to Ser393 in mouse), it’s not clear if mouse TAB1 is also only modified by O-GlcNAcylation on a single site or actually on multiple sites. The authors should clarify this point by experiments.
5. In Figure 3D, the authors showed that, as compared with WT TAB1, TAB1 ^S393A ^interacts normally with TAK1. More appropriate controls, such as the negative control antibody or treatment with LPS, should be included. For example, it has been reported that O-GlcNAcylation of TAB1 is induced upon IL-1 stimulation or osmotic stress. ^2^ It will provide better information if the experiments in Figure 3C and Figure 3D can be conducted in cells under stimulation or stress.
6. The authors claimed that this KI mouse line could be a good model for further study of the inflammatory diseases, but mouse brain tissues were used in the co-IP experiments in Figure 3. It will be more informative if immune cells from *Tab1* ^S393A ^mice can be included for comparison with WT immune cells. 

Is the work clearly and accurately presented and does it cite the current literature?

Yes

If applicable, is the statistical analysis and its interpretation appropriate?

Not applicable

Are all the source data underlying the results available to ensure full reproducibility?

Yes

Is the study design appropriate and is the work technically sound?

Partly

Are the conclusions drawn adequately supported by the results?

Partly

Are sufficient details of methods and analysis provided to allow replication by others?

Yes

Reviewer Expertise:

immunology

I confirm that I have read this submission and believe that I have an appropriate level of expertise to confirm that it is of an acceptable scientific standard, however I have significant reservations, as outlined above.

## References

[1] : Targeted disruption of the Tab1 gene causes embryonic lethality and defects in cardiovascular and lung morphogenesis. *Mech Dev*.2002;119(2) : 10.1016/s0925-4773(02)00391-x 239-49 10.1016/s0925-4773(02)00391-x 12464436

[2] : O-GlcNAcylation of TAB1 modulates TAK1-mediated cytokine release. *EMBO J*.2012;31(6) : 10.1038/emboj.2012.8 1394-404 10.1038/emboj.2012.8 22307082PMC3321193

# Response

**Published:** 2020-06-11

**Authors:** Daan van Aalten

**Affiliations:** University of Dundee, UK

We thank the referees for their critical reading of the manuscript and constructive feedback. Several points for improving the manuscript were made, in addition to good suggestions for further studies. Our aim of publishing this work in a rapid open access journal such as Wellcome Open Research was to report the availability of this mouse model, facilitating future research to dissect the role of this specific O-GlcNAc site on TAB1. As such, most of the experiments proposed are outside the scope of this manuscript and it is hoped our model will allow such experiments to be conducted in the future.

 Reviewer 1:

 1. Although the information was described in the Methods, the authors should also indicate the location of the restriction enzyme sites, primers and probes used in Figure 1. This will help the readers to understand the strategy. The authors should also provide the raw data of genomic sequencing containing the sequences encoding 393 Ala in KI and 393 Ser in WT mice.

 We agree that this suggestion would improve the readability of Figure 1. We have added the locations of restriction sites, primers and probes to the revised figure legend. We have provided sequences of wild type and mutant homozygous mice in an extra panel in Figure 2. 

 2. It has been reported that Tab1-deficiency caused embryonic lethality, due to the defects in lung and cardiovascular morphogenesis. ^1^ The authors showed that the heart weight of *Tab1* ^S393A ^mouse and WT mouse is comparable (Figure 3). It is unclear if there are any morphological abnormalities in the heart and lung of *Tab1* ^S393A ^mice. Histological analyses of heart and lung tissues should be done here. In addition, Figure 3B shows the heart weight is comparable between *Tab1* ^S393A ^and WT mouse at three months old. The unit in Y-axis should be clarified.

 This is a valuable suggestion. In the revised Figure 3 we have now data on normalised heart weights. We consider a detailed analysis of heart phenotypes as an interesting research avenue, but beyond the scope of this paper. 

 3. The title of this report is "A mouse model for functional dissection of TAB1 O-GlcNAcylation". To convince the readers that this mouse model can be used to dissect the function of TAB1 O-GlcNAcylation, the authors should demonstrate the potential biological effects of losing O-GlcNAcylation on TAB1 Ser393.  

 We have deliberated chosen the wording of this title to indicate that this manuscript reports the characterisation and validation (e.g. loss of O-GlcNAc) of the model, not a detailed functional dissection. We would prefer to leave the title as is.

 4. In Figure 3C, the authors used site-specific antibody to detect TAB1 O-GlcNAcylation in mouse cells. Although it has been reported that human TAB1 is O-GlcNAcylated on a single site at Ser395 (which is equivalent to Ser393 in mouse), it’s not clear if mouse TAB1 is also only modified by O-GlcNAcylation on a single site or actually on multiple sites. The authors should clarify this point by experiments.

 We have added (to Figure 1a) a sequence alignment of mouse and human TAB1 covering the 382-420aa of the C-terminal region that carries the TAB1 O-GlcNAc site. The human O-GlcNAc Ser residue is conserved in mouse and corresponds to the Ser393. We have clarified in the manuscript that the Ser393 residue is the sole reported O-GlcNAc site in mouse TAB1 *in vivo *according to previous studies (Trinidad *et al*. 2012; Alfaro *et al*. 2012) **. **We also replaced the mention of “single O-GlcNAcylation site” on mouse TAB1 “by sole reported O-GlcNAcylation site” from the manuscript. 

 5. In Figure 3D, the authors showed that, as compared with WT TAB1, TAB1 ^S393A ^interacts normally with TAK1. More appropriate controls, such as the negative control antibody or treatment with LPS, should be included. For example, it has been reported that O-GlcNAcylation of TAB1 is induced upon IL-1 stimulation or osmotic stress. ^2^ It will provide better information if the experiments in Figure 3C and Figure 3D can be conducted in cells under stimulation or stress.

 We have now added negative (IgG) and positive controls (macrophage lysates) to Figure 3 with the full blots deposited in the Figshare data repository along with the original unedited gels ( https://doi.org/10.6084/m9.figshare.9206567.v1 ( Authier, 2019a) ). We agree that the effects of loss of TAB1 O-GlcNAcylation can now be studied using this mouse model in different contexts, but this is beyond the scope of this paper.

 6. The authors claimed that this KI mouse line could be a good model for further study of the inflammatory diseases, but mouse brain tissues were used in the co-IP experiments in Figure 3. It will be more informative if immune cells from *Tab1* ^S393A ^mice can be included for comparison with WT immune cells. 

 We focused on brain tissues as they provided the largest amounts of TAB1 for biochemical studies.
